# KCNAB2 overexpression inhibits human non-small-cell lung cancer cell growth in vitro and in vivo

**DOI:** 10.1038/s41420-023-01679-5

**Published:** 2023-10-19

**Authors:** Feng Cheng, Yu-fei Tang, Yang Cao, Shi-qing Peng, Xiao-ren Zhu, Yue Sun, Shu-Hang Wang, Bin Wang, Yi-min Lu

**Affiliations:** 1 Department of Respiratory Medicine, Huzhou Central Hospital, Affiliated Central Hospital, Huzhou University, Huzhou, Zhejiang China; 2 Huzhou Key Laboratory of Precision Diagnosis and Treatment in Respiratory Diseases, Huzhou Central Hospital, Huzhou, Zhejiang China; 3 Department of Soochow Medical college, Soochow University, Suzhou, China; 4 Department of Respiratory, Affiliated Kunshan Hospital of Jiangsu University, Kunshan, China; 5 Clinical Research and Lab Center, Affiliated Kunshan Hospital of Jiangsu University, Kunshan, China

**Keywords:** Tumour biomarkers, Biomarkers

## Abstract

Non-small-cell lung cancer (NSCLC) accounts for approximately 85% of all lung cancer cases. NSCLC patients often have poor prognosis demanding urgent identification of novel biomarkers and potential therapeutic targets. KCNAB2 (regulatory beta subunit2 of voltage-gated potassium channel), encoding aldosterone reductase, plays a pivotal role in regulating potassium channel activity. In this research, we tested the expression of KCNAB2 as well as its potential functions in human NSCLC. Bioinformatics analysis shows that expression of *KCNAB2* mRNA is significantly downregulated in human NSCLC, correlating with poor overall survival. In addition, decreased KCNAB2 expression was detected in different NSCLC cell lines and local human NSCLC tissues. Exogenous overexpression of KCNAB2 potently suppressed growth, proliferation and motility of established human NSCLC cells and promoted NSCLC cells apoptosis. In contrast, CRISPR/Cas9-induced KCNAB2 knockout further promoted the malignant biological behaviors of NSCLC cells. Protein chip analysis in the KCNAB2-overexpressed NSCLC cells revealed that KCNAB2 plays a possible role in AKT-mTOR cascade activation. Indeed, AKT-mTOR signaling activation was potently inhibited following KCNAB2 overexpression in NSCLC cells. It was however augmented by KCNAB2 knockout. In vivo, the growth of subcutaneous KCNAB2-overexpressed A549 xenografts was significantly inhibited. Collectively, KCNAB2 could be a novel effective gene for prognosis prediction of NSCLC. Targeting KCNAB2 may lead to the development of advanced therapies.

## Introduction

Lung cancer is the second most common malignancy worldwide characterized by rapid progression and high invasiveness, which causes significant cancer-related mortalities every year worldwide. According to the 2020 global cancer research report, lung cancer takes up about 11.4% of all cancer cases while causing 18% of cancer-related death [1]. Further, among all lung cancers, approximately 85% are non-small-cell lung cancer (NSCLC) [2]. The current treatment strategies for NSCLC involve surgical resection, immunotherapy, and chemotherapy, which have failed to improve the overall survival as well as prognosis of patients with advanced NSCLC [3–6]. Hence, exploration of novel potential molecules for prognosis prediction as well as elucidating the underlying molecular mechanisms in NSCLC may be beneficial to developing potent targeted therapies for NSCLC patients.

Studies on ion channels have become the research focus in human cancers, where dysfunction of potassium channels is reported to affect the metabolic and angiogenic characteristics of cancer cells [7, 8]. The potassium ion channel is a membrane protein that drives the flow of potassium ions through an electrochemical gradient [7]. It plays a pivotal role in regulating neural signaling, preventing neuronal over-excitability and epithelial electrolyte transport. Recently, potassium channels have been reported to be involved in tumorigenesis including breast cancer [9], cervical carcinoma [10], non-Hodgkin lymphomas [11], and small-cell lung cancer [12]. KCNAB2 a member of the Kvβ superfamily, participates in building the Shaker (kv1) potassium channel complex together with KCNAB1 and KCNAB3 [13, 14]. KCNAB2 is located in band 3 zone 6 of the short arm of chromosome 1 [15]. Most of the studies on KCNAB2 have been focused on epilepsy [16, 17]. Yet, the biological functions of KCNAB2 in cancers are largely unknown. Only limited reports have studied the potential roles of KCNAB2 in peripheral T-cell lymphoma [18], neuroblastic lymphoma [19], and pituitary tumors [20]. Lee et al., have found that Kvβ2, encoded by KCNAB2, is overexpressed in alveolar epithelial cells while promoting the clearance rate of the alveolar fluid [21]. Lyu et al., have demonstrated that lung adenocarcinoma patients with low KCNAB2 expression have poor prognosis [22]. However, the expression of KCNAB2 and its potential functions in human NSCLC have not been studied yet. In the present study, we demonstrate an in vitro and in vivo inhibition of non-small-cell lung cancer (NSCLC) cell growth via exogenous overexpression of KCNAB2.

## Results

### KCNAB2 is down-regulated in human NSCLC

**Fig. 1:**
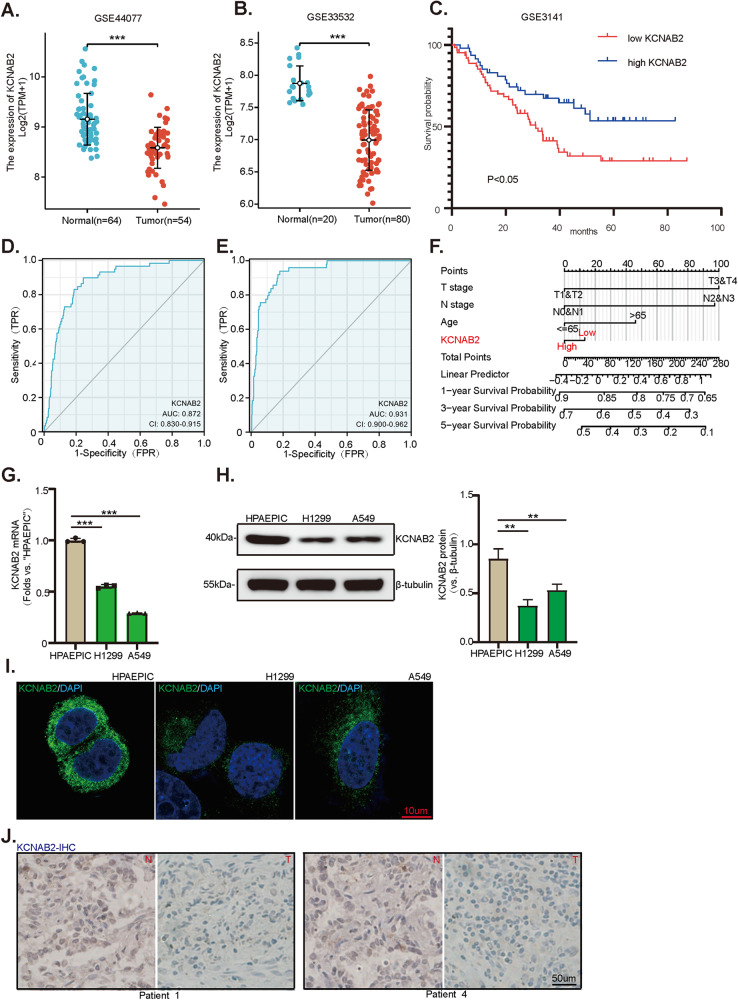
KCNAB2 is downregulated in human NSCLC. GEO databases (GEO44077, GEO33532) shows the *KCNAB2* mRNA transcripts in human NSCLC tissues and normal lung tissues (**A**, **B**). Kaplan Meier survival analyses of KCNAB2-low and KCNAB2-high NSCLC patients in GEO dataset (GSE3141) was presented (**C**). ROC curve was used to evaluate the predictive value of the KCNAB2 in lung adenocarcinoma (**D**) and lung squamous cell carcinoma (**E**). Calibration model was used to evaluate the value of KCNAB2 in predicting the prognosis of lung cancer patients (**F**). qRT-PCR and Western blotting were applied for testing the expression of KCNAB2 in NSCLC cells (A549 and H1299) and alveolar epithelial cells (HPAEPIC), with results quantified (**G**, **H**). KCNAB2 immunofluorescence images in the described cells were shown. Scale bar = 10 μm (**I**). The representative human tissue KCNAB2 IHC images were shown. Scale bar = 50 μm (**J**). A mean ± standard deviation (SD, n = 3) was used to represent the data. Results from three repeated experiments were obtained. *P < 0.05, ***P < 0.001.

First, GEO databases (GSE44077, GSE33532) reveal that KCNAB2 expression in human NSCLC tissues is significantly lower than it in the normal lung tissues (Fig. 1A, B). Next, Kaplan-Meier survival analysis of GEO dataset (GSE3141) shows that low KCNAB2 expression is correlated with poor prognosis of NSCLC patients (Fig. 1C). Receiver operating characteristic (ROC) curve results reveal that KCNAB2 downregulation has a significant predictive value on poor survival probability of patients with lung adenocarcinoma (Fig. 1D) and lung squamous cell carcinoma (Fig. 1E). Nomogram model suggests a good reliability of KCNAB2 in predicting prognosis of lung cancer patients (Fig. 1F).

To further validate the results of bioinformatics studies, we examined the expression of KCNAB2 in human NSCLC cells. The established human NSCLC cell lines (H1299 and A549) were cultured. qRT-PCR assay was performed for testing the expression of *KCNAB2* mRNA. As shown, *KCNAB2* mRNA expression in the established NSCLC cells was significantly lower than that in HPAEPIC (human alveolar epithelial cells) (Fig. 1G, *P* < 0.001). Western blotting and immunofluorescence dye further supported that the expression of KCNAB2 protein in NSCLC cells was significantly lower than that in HPAEPIC (Fig. 1H, I). Additionally, we tested the expression of KCNAB2 in local NSCLC tissues. We obtained NSCLC tissues and adjacent normal tissues from five primary NSCLC patients. The representative immunohistochemistry staining images of two representative patients (“Patient 1#”, “Patient 4#”) verified that KCNAB2 protein expression in the NSCLC tissues was significantly lower than that in the paired adjacent normal tissues (Fig. 1J). Together, these results confirmed that KCNAB2 is downregulated in human NSCLC.

### Exogenous overexpression of KCNAB2 inhibits NSCLC cell proliferation, migration and invasion

**Fig. 2:**
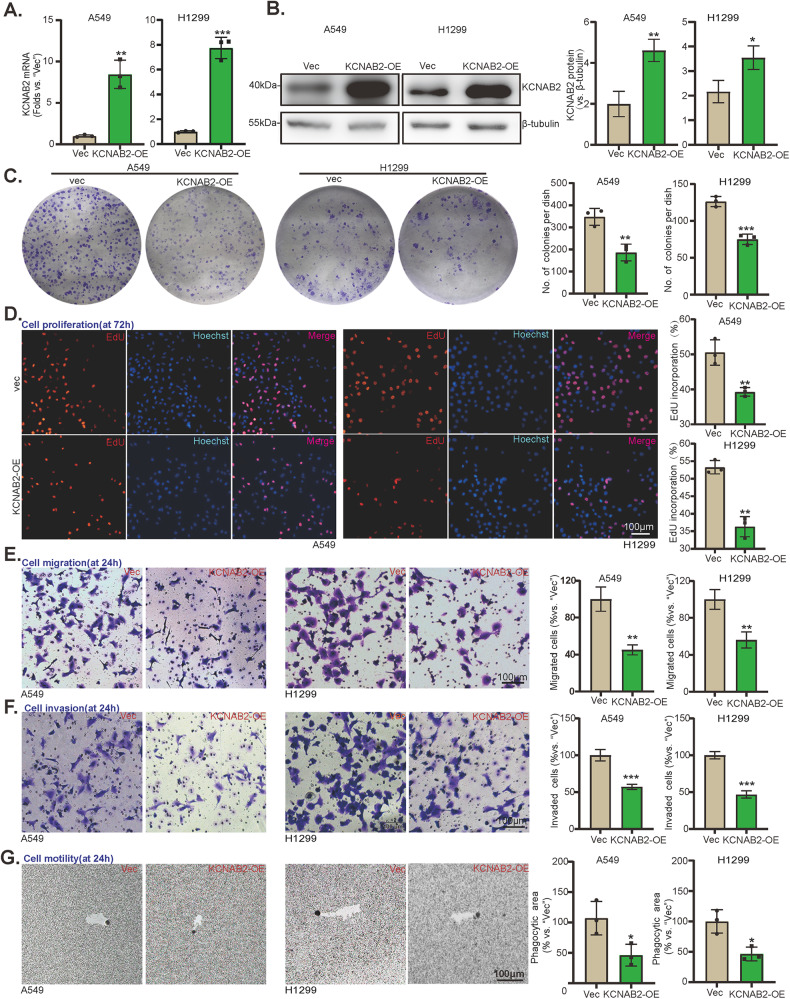
Exogenous overexpression of KCNAB2 inhibits NSCLC cell proliferation, migration and invasion. A549 and H1299 cells with KCNAB2 cDNA-expressing lentiviral construct (“KCNAB2-OE”) or empty vectors (“Vec”) were established. KCNAB2 expression among stable cells was measured via qRT-PCR (**A**) and Western blotting (**B**). Cell colony formation (**C**), proliferation (“EdU assay”, **D**), migration and invasion (“Transwell assays”, **E** and **F**), motility (phagokinetic track motility assays, (**G**) were examined. A mean ± standard deviation (SD, *n* = 3) was used to represent the data. Results from three repeated experiments were obtained. *P < 0.05, ***P < 0.001. Scale bar = 100 μm.

To investigate the potential effect of KCNAB2 on NSCLC cells, NSCLC cells (A549 and H1299) with the lentivirus encoding KCNAB2 cDNA (“KCNAB2-OE”) as well as the empty vector (“Vec”) were established. Puromycin was used for establishing stable cells. In order to test the KCNAB2 expression in stable cells, qRT-PCR and Western blotting were performed, and the results showed the expression of *KCNAB2* mRNA (Fig. 2A) and protein (Fig. 2B) increased significantly in KCNAB2-overexpressed (“KCNAB2-OE”) cells. Functional assays were conducted to determine the cell malignant behaviors. The results demonstrated that colony formation was potently inhibited in KCNAB2-overexpressed NSCLC cells (Fig. 2C). Furthermore, we have shown that the proliferation of NSCLC cells was potently inhibited via KCNAB2 overexpression as evident by a significantly decreased ratio of EdU-positive nuclei (Fig. 2D). Moreover, KCNAB2 overexpression largely suppressed in vitro migration (Fig. 2E), invasion (Fig. 2F), and motility (Fig. 2G) of NSCLC cells, tested by “Transwell”, “Matrigel Transwell” and phagokinetic track motility assays, respectively.

### Exogenous overexpression of KCNAB2 provokes apoptosis in NSCLC cancer cells

**Fig. 3:**
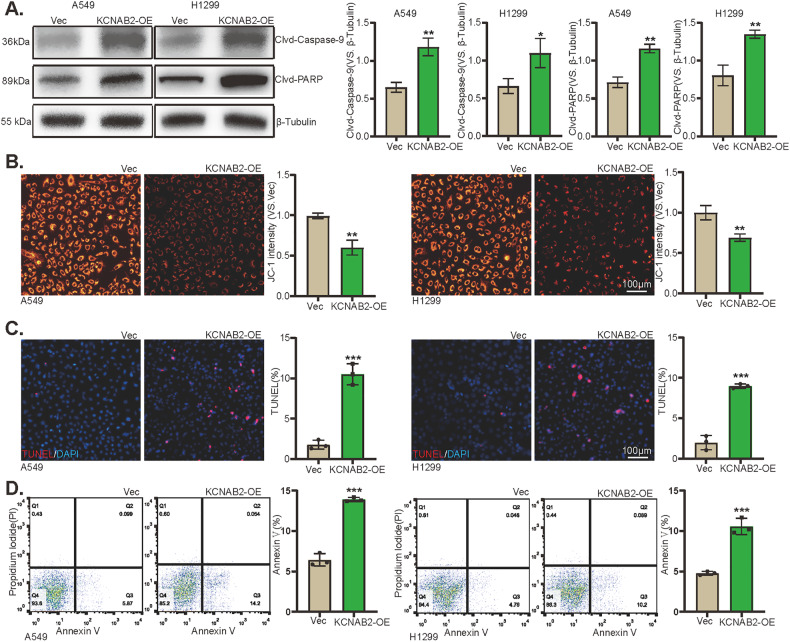
KCNAB2 overexpression provokes apoptosis in NSCLC cancer cells. The expression of listed proteins in the described cells was assessed via Western blotting assay (**A**), with the results quantified (**A**). Mitochondrial depolarization was detected by JC-1 staining assay (**B**). Cell apoptosis was detected by TUNEL staining (**C**) and Annexin V-PI FACS assays (**D**). A mean ± standard deviation (SD, *n* = 3) was used to represent the data. Results from three repeated experiments were obtained. “Clvd” stands for “cleaved”. *P < 0.05, **P < 0.01, ***P < 0.001. Scale bar = 100 μm.

Exogenous overexpression of KCNAB2 inhibited NSCLC cell malignant progression, we next investigated the effect of KCNAB2 overexpression on apoptosis of NSCLC cells. First we tested the caspase activities in KCNAB2*-*overexpressed (“KCNAB2-OE”) cells, results showed that cleavages of caspase-9 and PARP were significantly increased (Fig. 3A). Overexpression of KCNAB2 also leaded to depolarization of mitochondria, causing a decrease in membrane potential level (Fig. 3B). In addition, KCNAB2 overexpression increased the ratio of TUNEL-positive nuclei (Fig. 3C) and Annexin V-positively stained NSCLC cells (Fig. 3D), supporting apoptosis activation.

### KCNAB2-knockout promotes proliferation, migration as well as invasion of NSCLC cell

**Fig. 4:**
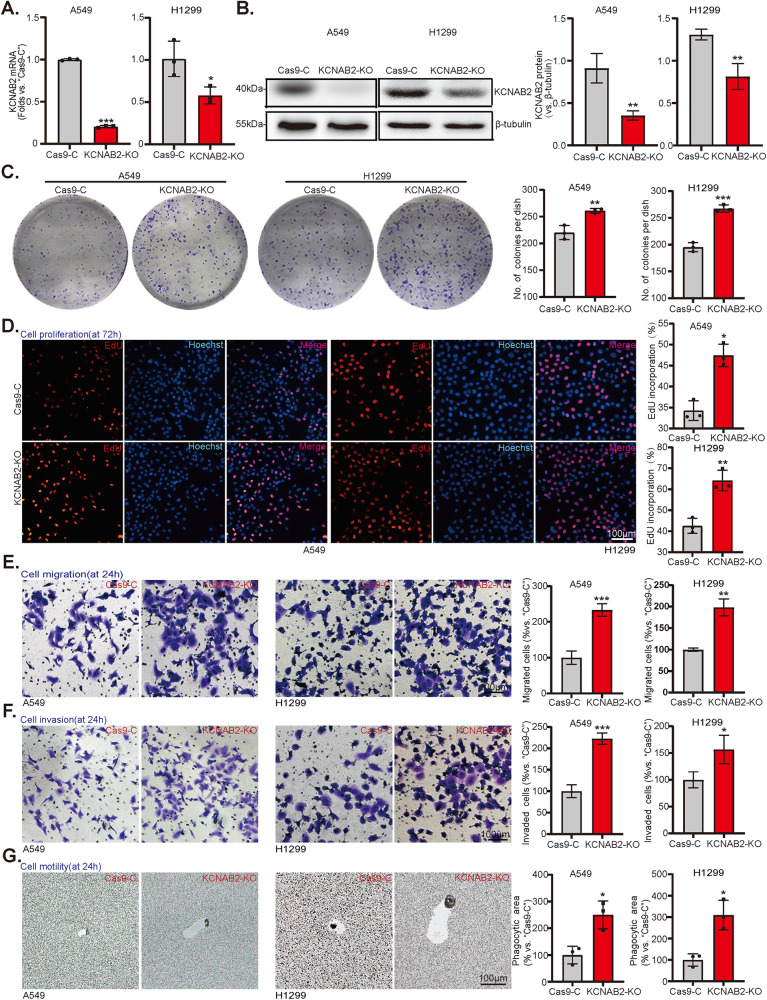
KCNAB2-knockout promotes proliferation, migration as well as invasion of NSCLC cell. A549 and H1299 cells with CRISPR/Cas9-KCNAB2-KO construct (“KCNAB2-KO”) or empty vector (“Cas9-C”) were established. KCNAB2 expression among stable cells was measured via via qRT-PCR (**A**) and Western blotting assays (**B**). The cell colony formation (**C**), proliferation (**D**), migration and invasion (**E** and **F**), and motility (**G**) were evaluated by assays as described, with data quantified. A mean ± standard deviation (SD, *n* = 3) was used to represent the data. Results from three repeated experiments were obtained. *P < 0.05, **P < 0.01, ***P < 0.001. Scale bar = 100 μm.

Besides we hypothesized that depletion of KCNAB2 could possibly exert pro-cancerous activity in NSCLC cell. The CRISPR/Cas9 gene-editing strategy was utilized. NSCLC cells (A549 and H1299) were transfected with the CRISPR/Cas9- KCNAB2-KO lentiviral constructs. The neomycin-based selection was used for establishing stable cells (“KCNAB2-KO”). As shown, the expression of *KCNAB2* mRNA and protein was substantially decreased in KCNAB2-KO NSCLC cells (Fig. 4A, B). Functional studies were performed as well, results showed that CRISPR/Cas9-mediated KCNAB2-KO promoted NSCLC cell colony formation (Fig. 4C), proliferation (Fig. 4D), migration (Fig. 4E), invasion (Fig. 4F), and motility (Fig. 4G), which were examined by cell colony formation, EdU staining, “Transwell”, “Matrigel Transwell” and phagokinetic track motility assays, respectively.

### KCNAB2-driven inhibition on NSCLC cell progression is partly mediated by inactivating AKT-mTOR cascade

**Fig. 5:**
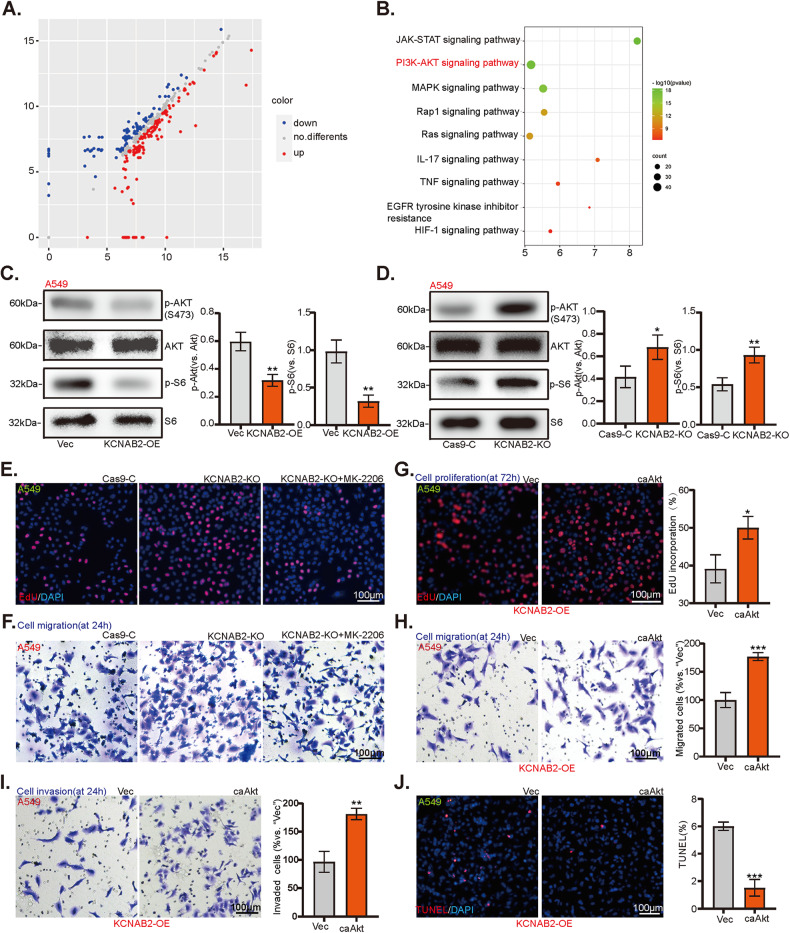
KCNAB2-driven inhibition on NSCLC cell progression is partly mediated by inactivating AKT-mTOR cascade. Differentially expressed proteins (**A**) and relative signaling pathways (**B**) were analyzed by protein chip analyses on KCNAB2-overexpressed NSCLC cells comparing vector control cells. The listed protein’s expression profile was measured (**C**, **D**), with the results quantified (**C**, **D**). Cell proliferation (“EdU assay”, **E**) and migration (“Transwell assay”, **F**) of KCNAB2-KO A549 cells treated with MK-2206 were detected. Cell proliferation, migration and invasion capacities were determined by conducting EdU-nuclei (**G**) and “Transwell” (**H**, **I**) assays on KCNAB2-OE A549 cells stably expressing constitutively active mutant Akt (S473D, “caAkt1”) or empty vector (“Vec”). Cell apoptosis was examined by TUNEL staining (**J**). A mean ± standard deviation (SD, *n* = 3) was used to represent the data. Results from three repeated experiments were obtained. *P < 0.05. Scale bar = 100 μm.

Next, we explored the possible underlying mechanisms of KCNAB2-driven inhibition on NSCLC cells. First, protein chip analysis was performed to analyze differentially expressed proteins (DEPs) in KCNAB2-overexpressed NSCLC cells compared with negative control cells (“Vec”), and KEGG enrichment analysis was performed subsequently. The results illustrated that DEPs in KCNAB2-overexpressed NSCLC cells were enriched in the regulation of multiple oncogenic pathways, among which phosphatidylinositol-3-kinase (PI3K)-AKT-mTOR cascade was most significant (Fig. 5A, B). AKT-mTOR signaling is one of the most important pro-cancerous cascades, and it is often hyper-activated in NSCLC [23–26]. The Western blotting results showed that KCNAB2 overexpression inhibited phosphorylation of AKT and S6 in NSCLC cells (Figs. 5C, S1A). Contrarily, AKT-mTOR cascade phosphorylation was augmented in the KCNAB2-KO NSCLC cells (Figs. 5D, S1B).

To explore whether AKT-mTOR activation was the primary cause of KCNAB2 KO-driven NSCLC cell progression, the AKT specific inhibitor MK-2206 was utilized. As shown, the proliferation (Figs. 5E, S1C) as well as migration (Figs. 5F, S1D) were inhibited by treatment with MK2206 in KCNAB2-KO NSCLC cells. Next, an adenovirus-packed sustained activated mutant Akt, caAkt1 (S473D), was stably transduced into KCNAB2 overexpressed NSCLC cells and it restore Akt phosphorylation (Fig. S1E). Functional assays showed that caAkt1 attenuated KCNAB2 overexpression-induced inhibition of proliferation (Figs. 5G, S1F), migration (Figs. 5H, S1G), and invasion (Fig. 5I, S1H) of NSCLC cells. In addition, caAkt1 reduced the TUNEL-positive nuclei ratio of KCNAB2 overexpressed NSCLC cells (Figs. 5J, S1I). Taken together, these results indicated that KCNAB2-driven suppression on NSCLC cell progression is partly mediated by inactivating AKT-mTOR cascade.

### KCNAB2 overexpression inhibited NSCLC cell growth in nude mice

**Fig. 6:**
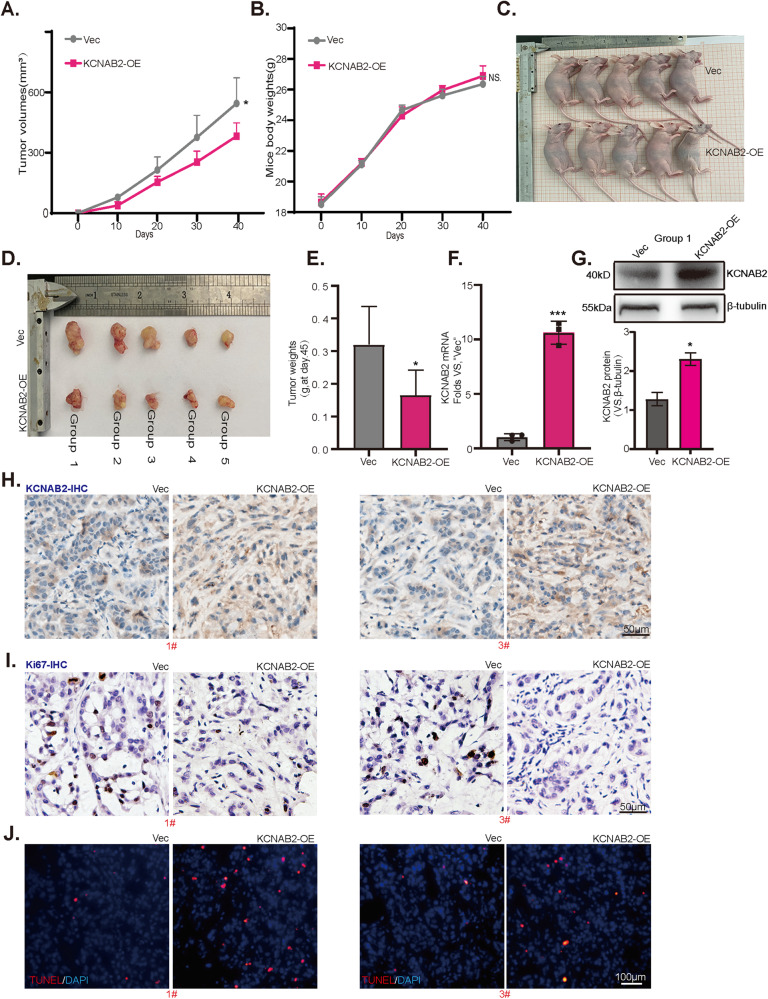
KCNAB2 overexpression inhibits NSCLC cell growth in vivo. The mice volumes (**A**) and body weights (**B**) were recorded every 10 days. Tumors were isolated (**C**, **D**) and weighed individually (**E**) after 40 days. Expression of *KCNAB2* mRNA and proteins in tumor tissue lysates were measured via qRT-PCR (**F**) and Western blotting (**G**), with data quantified. The representative IHC images of KCNAB2 (**H**) and Ki67 (**I**) in tumor tissues were showed, Scale bar = 50 μm. Representative TUNEL staining images in tumor tissues (**J**), Scale bar = 100 μm. A mean ± standard deviation (SD) was used to represent the data. *P < 0.05, ***P < 0.001.

Animal study was conducted to determine whether KCNAB2 could affect the growth of NSCLC cell in vivo. We subcutaneously injected equal amounts of KCNAB2-overexpressed A549 cells (“KCNAB2-OE”) along with vector control cells (“Vec”) in the right flanks of nude mice to establish tumor xenografts. Tumor volumes and weights were recorded every 10 days (Fig. 6A, B). Tumor growth curve results demonstrated that, as compared to control A549 xenografts, KCNAB2-overexpressed xenografts grew slower. At Day-40, xenografts of the two groups were isolated and weighed (Fig. 6C, D). As shown, KCNAB2-overexpressed xenografts were lighter than the control group (Fig. 6E). The mice body weights of the two groups were not significantly different. These findings demonstrated that KCNAB2 overexpression inhibited the growth of A549 xenograft in vivo.

Tumors from each group were separated and homogenized in fresh tissue lysis buffer. qRT-PCR along with Western blotting assays verified that as compared to the control groups, the expression of *KCNAB2* mRNA (Fig. 6F) and protein (Fig. 6G) was significantly upregulated in KCNAB2-overexpressed A549 xenografts. Moreover, in accordance with in vitro findings, the immunohistochemistry (IHC) staining results further supported that KCNAB2 overexpression in KCNAB2-OE xenograft slides (Fig. 6H). KCNAB2 overexpression inhibited the proliferation of A549 cells in vivo, evidenced by the decrease of Ki-67 staining (Fig. 6I). Furthermore, the xenograft slide immunofluorescence assay showed that TUNEL-positive nuclei ratio was significantly increased in KCNAB2*-*overexpressed A549 xenografts, supporting apoptosis activation (Fig. 6J).

## Discussion

The current study indicates that KCNAB2 overexpression inhibits human NSCLC cell growth. KCNAB2 is an important subunit and also an essential regulator of the KV1 channel complex [13, 14, 27]. The subtypes of the KV1 channel, such as KV1.1, KV1.3, and KV1.5, have been reported to participate in various biological processes in multiple malignancies [10, 28, 29]. The studies on KCNAB2 function are extremely limited. The underlying mechanisms of KCNAB2 potential functions are largely unknown either. Ashton et al., demonstrated that upregulation of KCNAB2 promoted cellular endocrine hormone secretion in somatotrophic pituitary adenoma [20]. White et al., have shown that the absence of *KCNAB2* gene in specific regions is associated with neuroblastoma development [19]. The expression of KCNAB2 and its potential functional role in NSCLC and other human malignancies have not yet been investigated.

The results of the present study suggested that KCNAB2 should be a vital gene for NSCLC cell growth. GEO database shows that *KCNAB2* mRNA transcripts are significantly downregulated in human NSCLC tissues, and low-KCNAB2 expression in NSCLC correlates with the poor overall survival of NSCLC patients. *KCNAB2* mRNA and protein expression is significantly downregulated in immortalized NSCLC cell lines as well as in local NSCLC tissues when compared with human alveolar epithelial cells and corresponding adjacent normal tissues. Lyu et al., have shown that KCNAB2 expression is downregulated in lung adenocarcinoma [22]. The proliferation and migration ability of lung cancer cells was altered after KCNAB2 overexpression by plasmid transfection, although the difference is not statistically significant [22]. However, in this study we confirmed that in different NSCLC cells, ectopic overexpression of KCNAB2 potently inhibited the cell malignant biological behaviors. Contrarily, CRISPR/Cas9-induced KCNAB2 KO accelerated the growth, proliferation, migration, and invasion of NSCLC cells. In vivo the growth of KCNAB2-OE A549 xenografts was significantly suppressed.

AKT-mTOR signaling pathway is a major growth regulatory signaling pathways in various cancers [30] and regulates various important biological processes, including metabolism and proliferation of the cell, progression of cell cycle and cell survival, apoptosis resistance, and genomic instability [31–35]. Over-activation of this cascade is frequently detected in NSCLC and it is associated with poor prognosis [23–26]. Previous studies have demonstrated that increased potassium channel activity mediated by potassium ion can suppress the malignant growth behavior of NSCLC cells through inhibition of the AKT-mTOR pathway [36–39].

In this study, protein chip analysis revealed that the AKT-mTOR signaling activation is inhibited in KCNAB2-overexpressed human NSCLC cells. Importantly, Western blotting results demonstrated remarkable inhibition of AKT-mTOR activation in KCNAB2-overexpressed A549 and H1229 cells. Furthermore, Ki67 decrease was also detected in KCNAB2-overexpressed A549 xenograft tumor tissues. The AKT inhibitor inhibited KCNAB2-KO NSCLC cell proliferation and migration. Taken together, our findings indicate that KCNAB2 overexpression-driven NSCLC cell inhibition should be partly via the inactivation of AKT-mTOR cascade. However, the mechanisms warrant further investigations.

## Conclusion

KCNAB2 overexpression inhibits human NSCLC cell growth in vitro and vivo.

## Materials and methods

### Human tissues and cells

The NSCLC tissues together with adjacent lung tissues derived from five written-informed consent primary patients were obtained from Affiliated Kunshan Hospital of Jiangsu University. Written-informed consent was obtained from all of the patients. According to the Declaration of Helsinki, the Ethics Committee of Jiangsu University (BR2015021) has approved this study. Established NSCLC cell lines (A549 and H1299) and alveolar epithelial cells (HPAEPIC) were purchased from the Cell Bank at the Shanghai Institute of Biological Science (Shanghai, China). These cell lines were cultured in DMEM/F-12 medium plus 10% FBS (Gibco, Suzhou, China) and incubated in a 37 °C incubator with a 5% CO_2_ humidified atmosphere.

### KCNAB2 overexpression

The KCNAB2-expressing lentiviral vector (“LV-KCNAB2”) and the negative control lentiviral vector were provided by Genechem (Shanghai, China). The wells of the 6-well plate were seeded with NSCLC cells using 1 × 10^5^ cells per well. When the cell fusion rate reached 60% in each well, the lentivirus (MOI = 10) was added to cell culture medium. Stable KCNAB2-overexpressed cells were established with puromycin (3 µg/ml). KCNAB2 expression among stable cells was finally determined via qRT-PCR and Western blotting assays.

### KCNAB2 knockout

A lenti-CRISPR/Cas9-GFP-puro construct containing the small guide RNA (sgRNA) targeting human KCNAB2 and the negative control construct were provided by Genechem (Shanghai, China) and added in cultured human NSCLC cells. KCNAB2 KO stable cells were selectively grown with neomycin for a duration of seven days and were then tested via Western blotting and qRT-PCR assays.

### Constitutively-active mutant Akt1

The constitutively-active mutant Akt1 (caAkt1 S473D) was packaged through lentivirus [40], which was transduced to cultured KCNAB2-OE NSCLC cells. Stable cells were established with neomycin-mediated selection. caAkt1 expression among stable cells were measured by Western blotting.

### qRT-PCR

Total RNA was extracted by the RNA rapid extraction kit (Yishan, Shanghai, China) and was reversely transcripted to cDNA via RT easy quick kit (Jiangsu CoWin Biotech Co, Ltd, Suzhou, China). It was then used for performing PCR experiments with an SYBR Green PCR Master Mix. Calculation of the targeted mRNA quantification was done via the 2^^-∆∆Ct^ method. Glyceraldehyde-3-phosphate dehydrogenase (GAPDH) was examined as an internal control. The primers were verified and provided by Genechem (Shanghai, China).

### Western blotting

In brief, the same set of lysates (20 µg aliquots) was run on 10% SDS gels. Different proteins with different molecular weights were separated and transferred to polyvinylidene difluoride (PVDF) membranes (Millipore, Bedford, MA). The membranes were then blocked with skim milk (10%) and incubated with following indicated primary antibodies: KCNAB2 (#orb324737, biorbyt, 1:2500 dilution), Tubulin (#ab179513, Abcam, 1:5000 dilution), anti-Akt (#4060, Cell Signaling Technology, 1:3500 dilution), anti-S6 (#2217, Cell Signaling Technology, 1:3500 dilution), anti-phospho-S6 (#ab12864, Abcam, 1:3500 dilution), anti-phospho-Akt (S473) (#4060, Cell Signaling Technology, 1:3500 dilution), anti-cleaved-poly (ADP-ribose) polymerase (PARP) E51 (#ab32064, Abcam, 1:1500 dilution), anti-cleaved-Caspase 9 (#ab2324, Abcam, 1:1500 dilution) overnight at 4 °C. An enhanced chemiluminescence kit (ECL, Millipore, Schwalbach, Germany) was used to visualize the blots after incubating membranes with HRP-conjugated secondary antibodies for 2–4 h at 25 °C. The intensity of bands was measured via Image J software.

### Immunohistochemistry (IHC)

IHC staining was carried out on paraffin-embedded tissue sections. Briefly, after dewaxing, the tissue slices (3 µm) were covered with diluted primary antibodies and incubated at 4 °C in a humidified box overnight. A half-hour incubation with secondary antibody was performed after slices were washed with PBS. Last, slices were photographed using an FSX100 microscope (Olympus, Tokyo, Japan).

### Cellular immunofluorescence assay

NSCLC cells and alveolar epithelial cells were seeded on glass coverslips. Cells were cultured at 50–60% confluence and then incubated with KCNAB2 primary antibody overnight, followed with incubation with green fluorescent secondary antibody for 2 h. Finally, cell nucleus was stained with DAPI (Invitrogen), Fluorescence images were photographed under a confocal fluorescence microscopy (Zeiss, Germany).

### Phagokinetic track motility assay

Adding 1 ml of coating medium of fibronectin (20 μg/ml) to the chambers of 12-well plate and let stand for 2 h. In addition, wells were added with 1.2 ml of microsphere suspension (43 µL of stock microbeads solution diluted in 15 ml of DMEM/F12). The plates were then centrifuged at 1,200 r for 20 min and placed into a 37 °C incubator for 1 h. The supernatant (2 ml) was removed from each well and cells were freshly re-suspended in 2 ml of conditioning medium (DMEM/F12 plus 0.05% FBS) seeded into each well (800 cells per well). Culturing of cells was done for 24 h, and imaged using an Olympus FSX100 microscope.

### Other assays

Cell colony formation, cell migration and invasion assays, EdU staining assay, mitochondrial depolarization assay and Annexin V-PI flow cytometry were described in detail in our previous studies [41, 42].

### Xenograft assay

The animal procedures were approved by IACUC and the Ethics Review Board of Jiangsu University. The 5–6 week-old female nude mice (18–19 g) were purchased from SLAC (Shanghai, China). Maintenance of nude mice was done under standard conditions. For constructing the xenograft model, we injected A549 cells with/without applied genetic modifications (1 × 10^6^ cells per mouse in 10% FBS F12K/Matrigel solution) subcutaneously into the right flanks of the mice. Volumes as well as body weights of the tumor in mice were recorded every 10 days. The resection of tumors was done 40 days later and tumor weights were recorded for additional experiments.

### Statistical analysis

The in vitro experiments for this study were done in triplicates. We conducted the statistical analysis and drew charts using SPSS 22.0 and GraphPad prism 7.0. Experimental data were presented as the mean ± standard deviation (SD). A two-tailed unpaired student’s *t*-test and one-way ANOVA along with Scheffe’ and Tukey Test were applied to compare the significance across different groups. We used *P* < 0.05 as the criterion for a statistically significant outcome.

### Supplementary information

Original data set

Figure Supplementary materials 1

## Data Availability

The data presented in this study is available on reasonable request from the corresponding author.
