# Supplementary figures and images for: KCNAB2 overexpression inhibits human non-small-cell lung cancer cell growth in vitro and in vivo
Source: Cell Death Discov. 2023 Oct 19;9:382. doi: 10.1038/s41420-023-01679-5 (PMC10584983; doi:10.1038/s41420-023-01679-5)

Figure 1

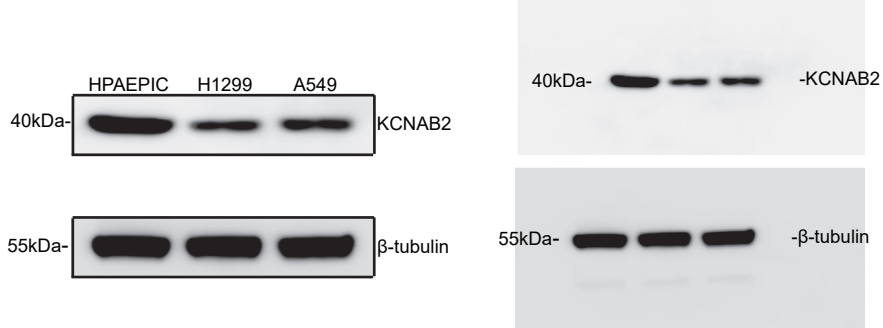

Figure 2

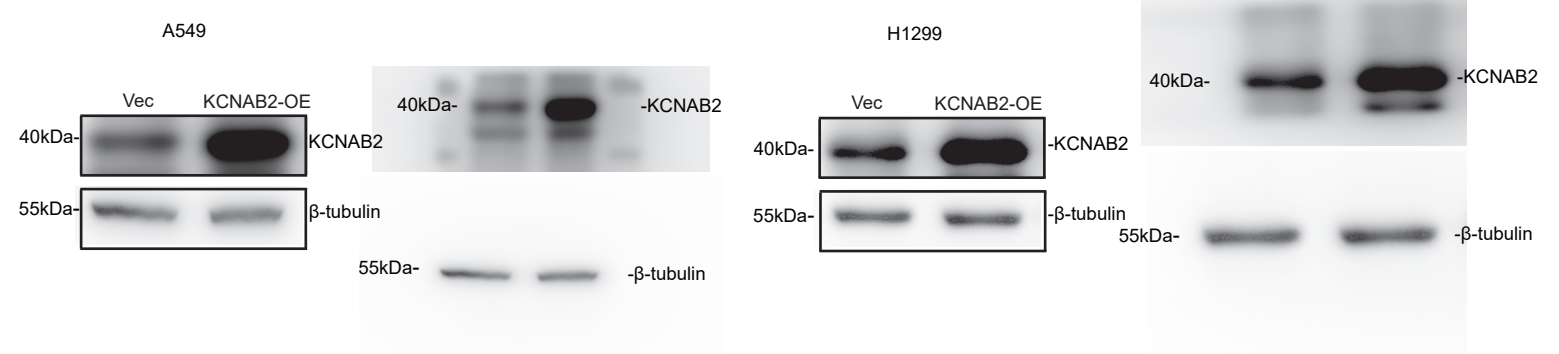

Figure 3

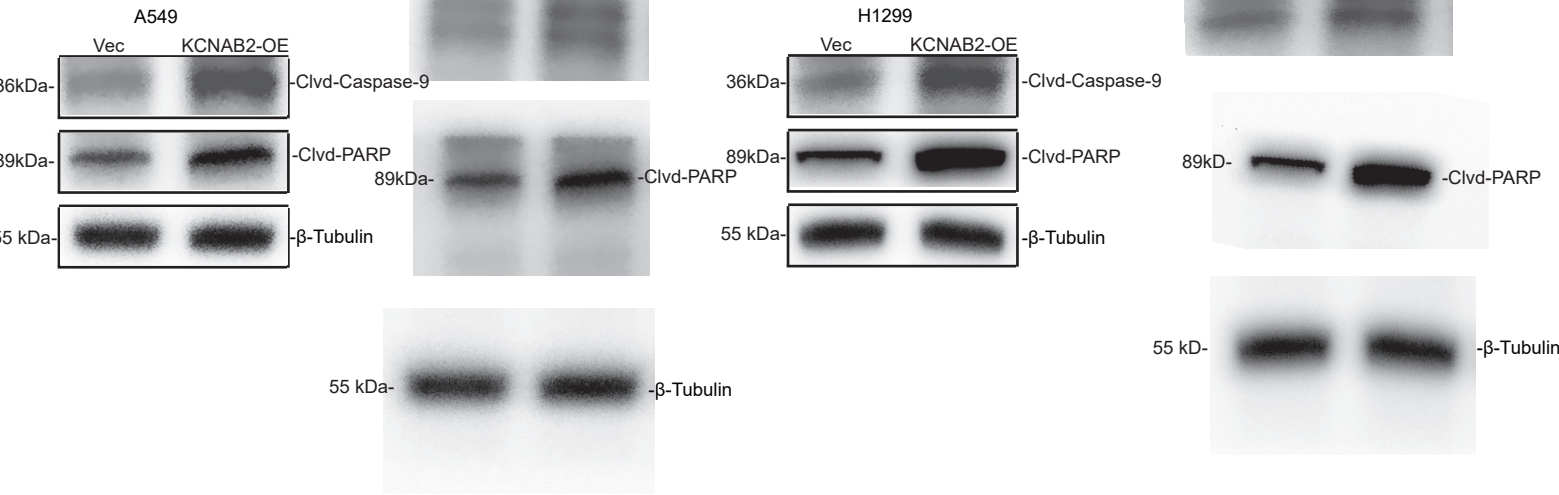

Figure 4

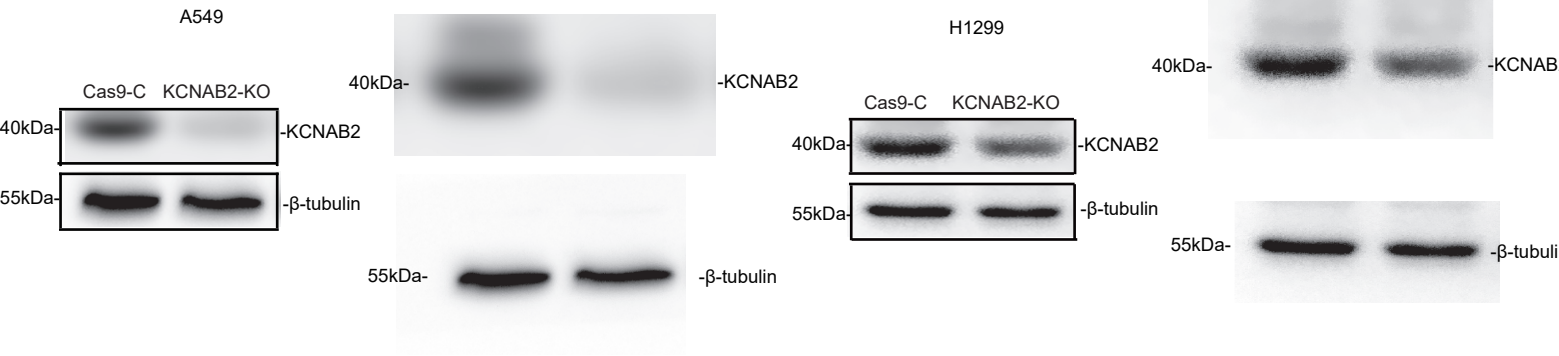

Figure 5

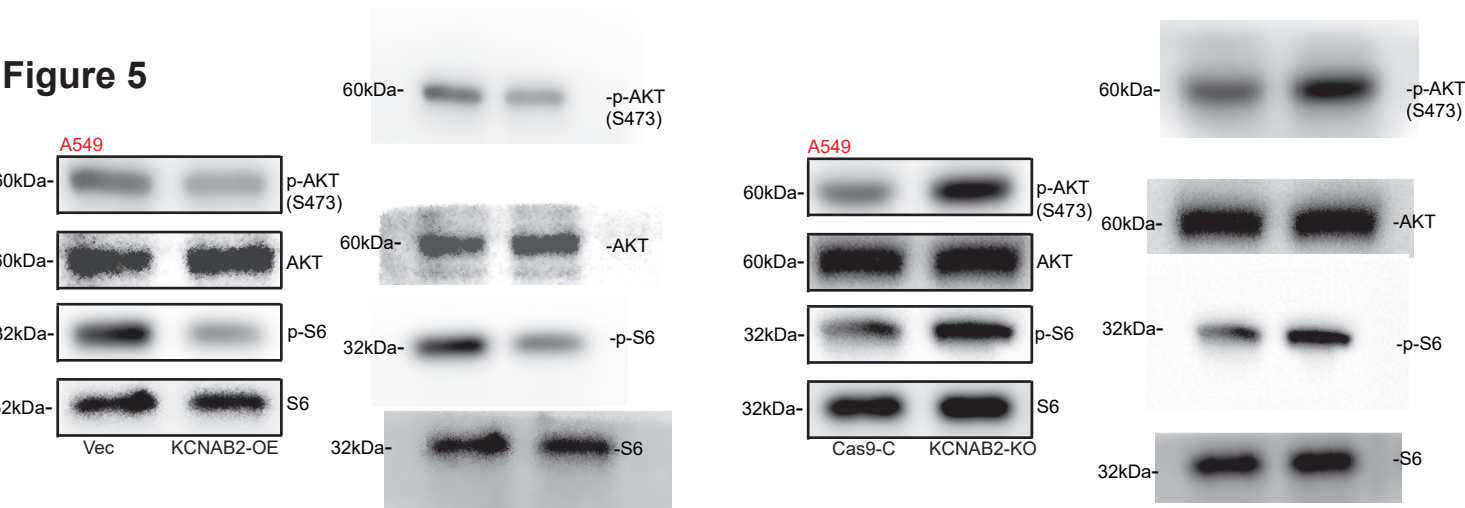

Figure 6

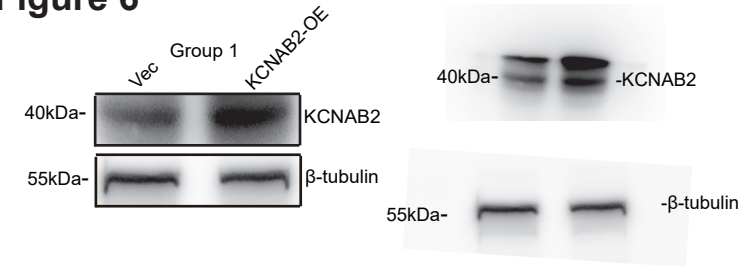

Figure Supplementary materials 1

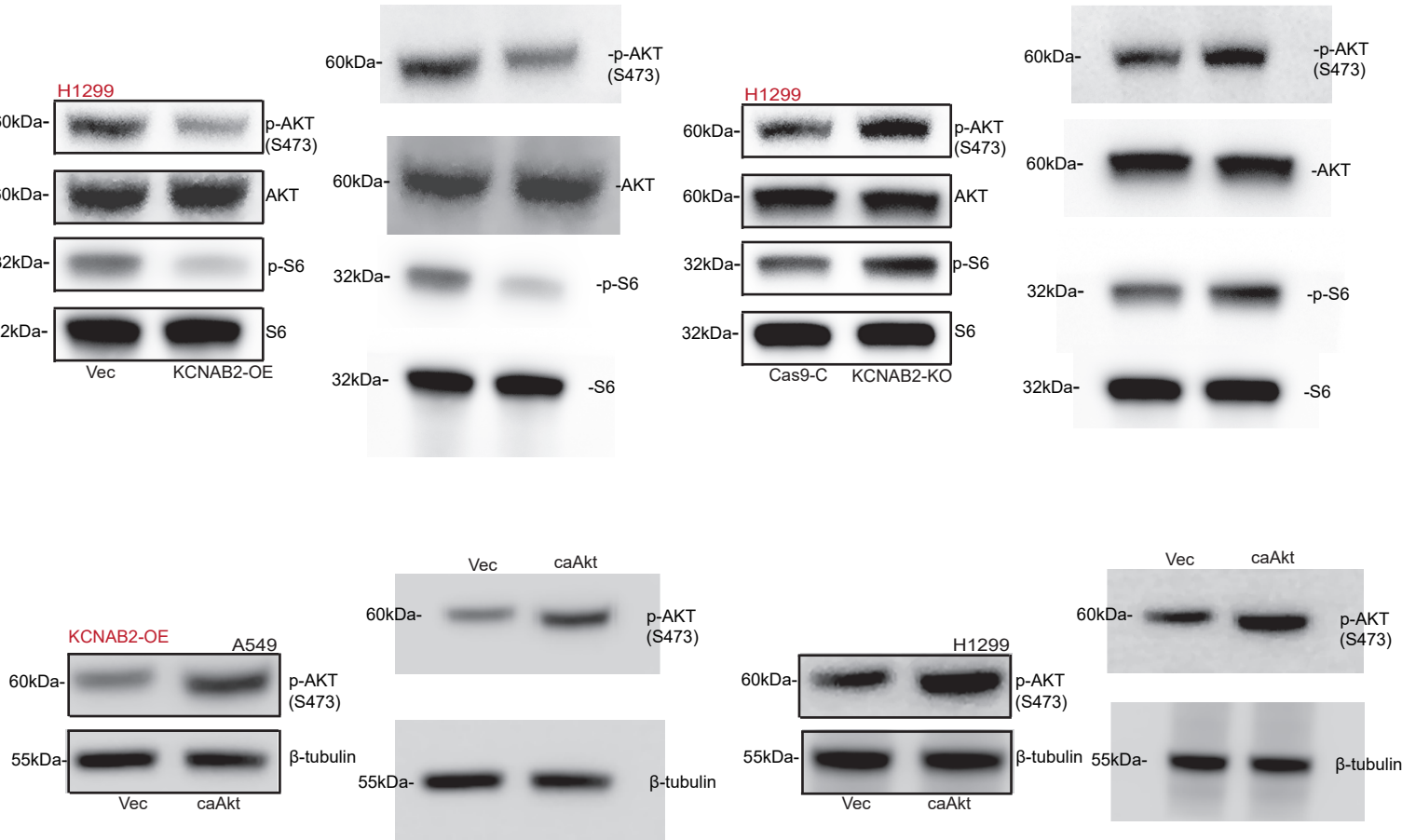

Supplement: Supplementary file 1 — Original data set [file 41420_2023_1679_MOESM1_ESM.pdf]
