# Supplementary material for: KCNAB2 overexpression inhibits human non-small-cell lung cancer cell growth in vitro and in vivo
Source: Cell Death Discov. 2023 Oct 19;9:382. doi: 10.1038/s41420-023-01679-5 (PMC10584983; doi:10.1038/s41420-023-01679-5)
Supplement: Supplementary file 2 — Figure Supplementary materials 1 [file 41420_2023_1679_MOESM2_ESM.pdf]

Figure S1

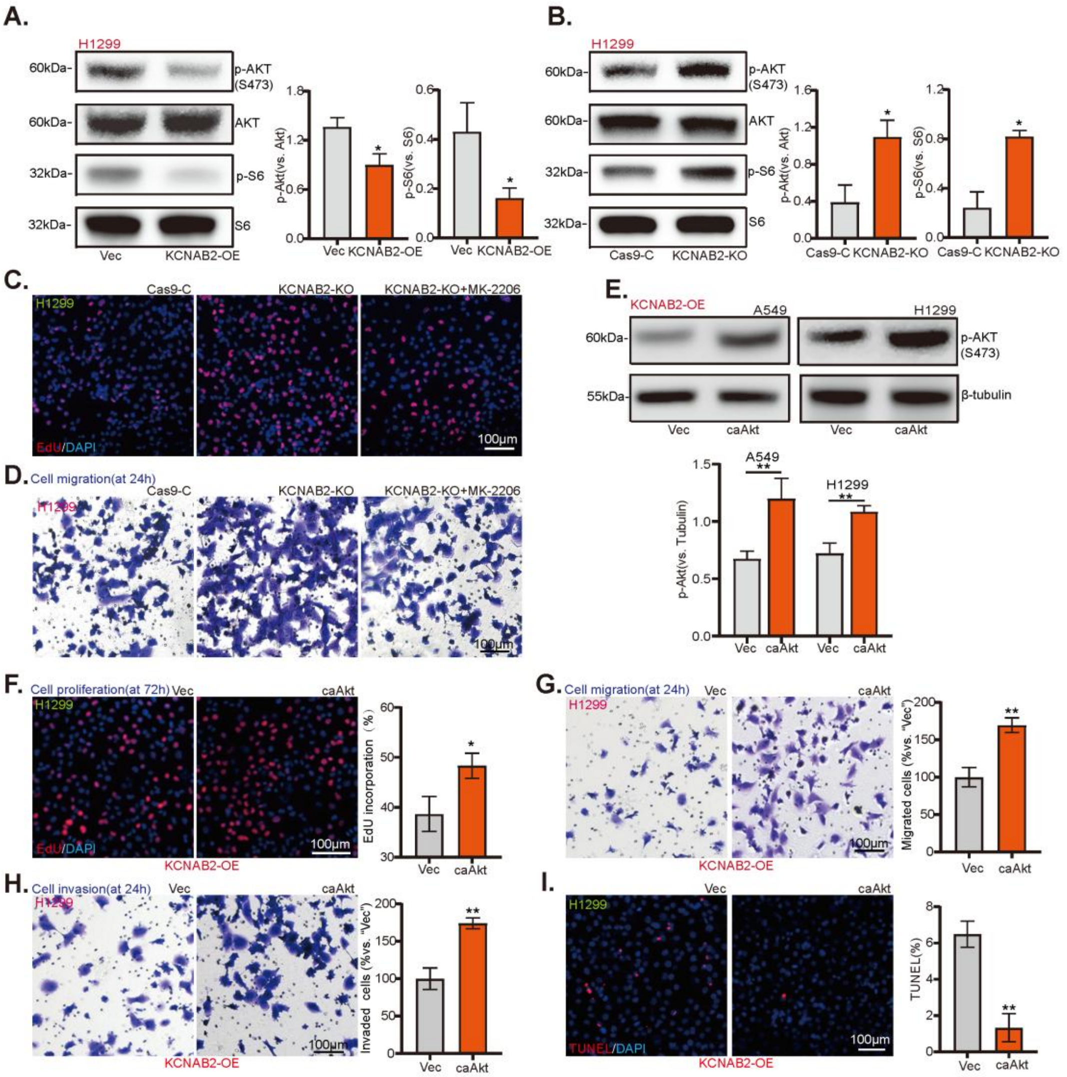

Figure S1. Stable NSCLC cell lines with the lentiviral KCNAB2 cDNA ("KCNAB2-OE"), the CRISPR/Cas9-KCNAB2-KO ("KCNAB2-KO") construct, the corresponding control empty construct were established. KCNAB2-KO were also treated with or without MK-2206 and KCNAB2-OE cells were stably transduced with caAkt1 construct or the empty vector. The expression of the listed proteins was detected (**A** and **B**), with the results quantified (**A** and **B**). Cell proliferation ("EdU assay", **C**) and migration ("Transwell assay", **D**) were examined. AKT phosphorylation in stable cells was measured (**E**), with data quantified (**E**). Cell proliferation ("EdU assay", **F**), migration and invasion ("Transwell" assays, **G** and **H**) as well as cell apoptosis (TUNEL staining, **I**) were tested. A mean ± standard deviation (SD, n=3) was used to represent the data. Results from three repeated experiments were obtained. *$P < 0.05$. Scale bar = 100 μm.
